# Ecological risk assessment and heavy metals accumulation in agriculture soils irrigated with treated wastewater effluent, river water, and well water combined with chemical fertilizers

**DOI:** 10.1016/j.heliyon.2023.e14580

**Published:** 2023-03-16

**Authors:** Hamed Soleimani, Borhan Mansouri, Amir Kiani, Abdullah Khalid Omer, Moslem Tazik, Gholamreza Ebrahimzadeh, Kiomars Sharafi

**Affiliations:** a Department of Environmental Health Engineering, School of Public Health, Tehran University of Medical Sciences, Tehran, Iran; b Student's Scientific Research Center, Tehran University of Medical Sciences, Tehran, Iran; c Substance Abuse Prevention Research Center, Research Institute for Health, Kermanshah University of Medical Sciences, Kermanshah, Iran; d Regenerative Medicine Research Center (RMRC), Kermanshah University of Medical Sciences, Kermanshah, Iran; e Pharmaceutical Sciences Research Center, Health Institute, Kermanshah University of Medical Sciences, Kermanshah, Iran; f Department of Food Hygiene and Quality Control, Faculty of Veterinary Medicine, Urmia University, Urmia, Iran; g Department of Environmental Health Engineering, School of Public Health, Zabol University of Medical Sciences, Zabol, Iran; h Research Center for Environmental Determinants of Health (RCEDH), Research Institute for Health, Kermanshah University of Medical Sciences, Kermanshah, Iran; i Department of Environmental Health Engineering, School of Public Health, Kermanshah University of Medical Sciences, Kermanshah, Iran

**Keywords:** Soil pollution, Heavy metals, Irrigation resources, Ecological risk assessment, Agriculture, Water reuse

## Abstract

Contaminated irrigation water can increase trace heavy metals concentration in agricultural soil. The present research aimed to investigate the effect of three types of irrigation water sources, including treated wastewater effluent, *Gharasoo* river water, and well water with chemical fertilizer, on the accumulation and ecological risk of heavy metals in agricultural soils. Soil samples were collected before and after crop irrigation to evaluate heavy metal concentrations. The samples were analyzed to determine the presence of arsenic, nickel, cadmium, iron, chromium, zinc, lead, copper, and manganese. Based on the results, the concentration of essential metals in the soil before the irrigation process was more than toxic metals. The different irrigation sources increased the concentration of all heavy metals in the soil, and the accumulation of Cr, Ni, and Cd significantly elevated more than others. Irrigation resources' effectiveness in transferring heavy metals to the soil was obtained as treated wastewater effluent < well water with fertilizer < river water. Furthermore, the potential ecological risk index (RI) for irrigated soil was in a high-risk category. Therefore, it is recommended that the river water should not be used to irrigate vegetables to the utmost possible. Finally, the low heavy metals concentration and the presence of nutrients in treated wastewater effluent make this source the most suited source of irrigation because it eliminates the need for chemical fertilizers by farmers and transfers fewer heavy metals to the soil.

## Graphical abstract

**Figure:**
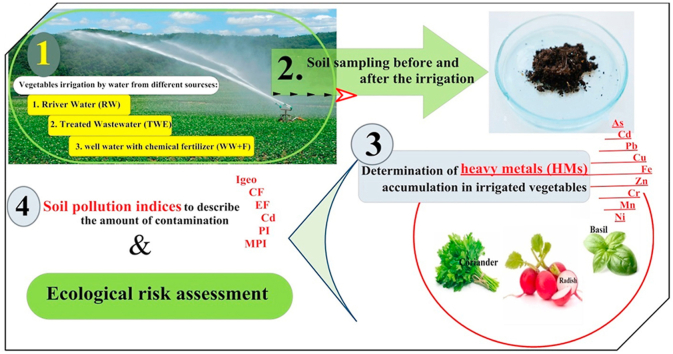

## 1 Introduction

Today, water resource shortage has been taking place everywhere in the world. The limitation of freshwater resources has attracted researchers and officials to use unconventional water, such as saltwater, rainwater, and domestic and industrial wastewater [[1], [2], [3]]. Physical soil qualities, fertility, soil structure, and organic matter content can all be enhanced by irrigation with wastewater that has been properly treated [4]. So, large-scale wastewater reuse projects, especially municipal wastewater, are being implemented in industrialized and developing countries [5].

An alternative source to compensate for the lack of fresh water is treated domestic wastewater used in agricultural irrigation. Untreated and treated wastewater irrigates about 20 Mha worldwide [6]. The combination of domestic wastewater effluents and water supply can meet the nutritional needs of plants. Nevertheless, an important point that should be considered is investigating the health and ecological effects of treated domestic wastewater use on cultivated crops' soil and physical and chemical properties [7].

The main problem of crop irrigation with wastewater is heavy metals in wastewater, which might deposit in the soil and eventually end up in cultivated plants [8]. Prolonged usage of wastewater effluents for irrigation often increases soil heavy metal content [9]. Toxic metal buildup decreases soil fertility and crop quality while interfering with the soil's ecological function and effects on other ecosystem elements. Furthermore, HMs can be released as solutions, which the rhizosphere of the plants can then absorb when the soil's capacity to contain heavy metals declines due to a rise in HMs at the soil level [10].

On the other hand, using treated wastewater instead of groundwater for irrigation purposes in arid and semi-arid regions is crucial since it boosts treated wastewater's reusability and decreases the need for agrochemicals [11,12]. Treated wastewater is more cost-effective than groundwater and chemical fertilizers, following all associated standards. Moreover, controlling and monitoring contaminants added to the soil, including heavy metals, is necessary through irrigation with these alternative sources [13].

The provision of irrigation water is one of the serious concerns being debated by provincial officials in Kermanshah Province, which is one of the agricultural centers of Iran. Due to the limited groundwater resources in Iran, including Kermanshah province, it is necessary to look into additional water supplies for agricultural irrigation. The water from the Gharasoo River and purified wastewater effluent from the local wastewater treatment plants are two of the most effective alternatives to groundwater for agricultural irrigation.

The comparative evaluation of the effects of three different irrigation sources on agricultural soil contamination in heavy metals is one of this research's essential innovations and novelties. Studies with this methodology are minimal everywhere in the world. Most previous research focused on one irrigation source, and this procedure cannot determine the best source of irrigation because their characteristics vary [[13], [14], [15]]. The current study intended to address prior studies' limitations by employing the same irrigation source, irrigation time, irrigation period, vegetable variety, and soil physical and chemical features. An additional novel aspect of this study is the difficulties farmers and responsible organizations face in Iran while determining the best irrigation method for crop cultivation. In this study, we also tried to figure out how irrigation water, such as well water with chemical fertilizers (WWF), treated wastewater effluent (TWE), and river water (RW), affects the number of heavy metals in soil vegetable culture. These metals include essential metals (including iron (Fe), manganese (Mn), copper (Cu), zinc (Zn)) and non-essential metals (including arsenic (As), lead (Pb), cadmium (Cd), nickel (Ni), and chromium (Cr)). The assessment of HMs in soil could be evaluated by various quantitative pollution indices such as the geoaccumulation index (Igeo). These indices are an objective tool for assessing the natural enrichment of soils with trace elements. Finally, the potential ecological risk index (RI) was used to quantify ecological risk in soils irrigated with various sources.

## 2 Material and methods

### 2.1 Study area

The required cultivation site was prepared by coordinating and consultations with the Kermanshah Municipal Wastewater Treatment Plant. Kermanshah province in Iran is 24,640 km^2^ in size and is located between 33°37′-35°17′N and 45°20′-48°1′E. The annual precipitation average is 450 mm. Summers in Kermanshah are hot, arid, and clear, while winters are cold and partly cloudy. Throughout the year, the temperature typically ranges from −4 °C to 38 °C, rarely falling below −8 °C or rising above 44 °C.

### 2.2 Crop growth conditions and irrigation treatments

**Fig. 1:**
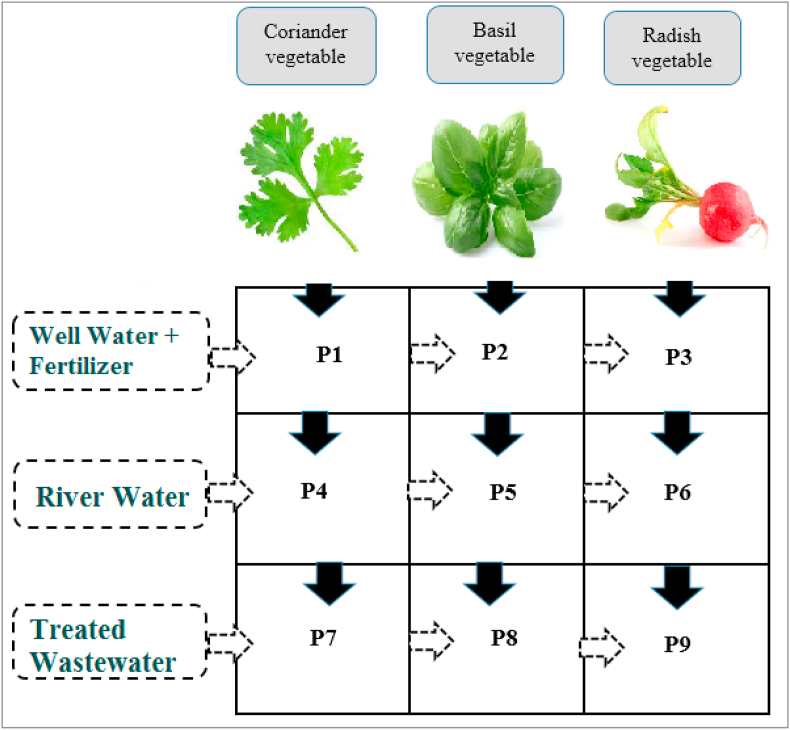
The pattern of designated plots for the cultivation of vegetables and different irrigation types of water sources.

A 36-square-meter piece of agricultural land near Kermanshah's wastewater treatment facility was chosen for this study and split into nine portions (P) equal to 2*2 (4 square meters) (Fig. 1). Three crop species are grown: coriander in (P1, P4, P7) parts, basil in (P2, P5, P8) parts, and radish in (P3, P6, P9) parts. Three distinct water sources (TWE, RW, and WW + F) were used to irrigate these veggies. In addition, each farmed land section was analyzed for certain products with a specific irrigation water supply. Three kinds of irrigation sources have been indicated in Table 1.

**Table 1:** Irrigation sources and cultivated portions.

Irrigation source	Farmed field portions
Well water (groundwater) with fertilizer (WWF)	P1, P2, and P3
*Gharasoo* river water (RW) watered	P4, P5, and P6
Treated wastewater effluent (TWE)	P7, P8, and P9

All nine farmed fields (Fig. 1 & Table 1) were initially irrigated with well water after ploughing and seeding, then once every three days for two months (60 days). There were 20 phases of irrigation for each of the several irrigation sources. All areas were also irrigated after sundown when the temperature was ideal for irrigation. The International Commission on Irrigation and Drainage's standard guidelines for water utilization in irrigation systems have been adopted (ICID) in this study [16,17].

### 2.3 Sampling

#### 2.3.1 Plant and soil sampling

Before cultivation, the soil's primary properties were assessed to ensure contamination had not happened before watering. In this regard, the HMs content of the soils was measured by taking multiple composite samples (from 5 points, including P1, P2, P7, P9, and P5) from the top layer soil (30 cm). The pH of the initial soil varied between 7.3 and 7.5. The measured HMs content of the soil was lower than the announced standards [8]. The principal soil properties prior to irrigation Table 5.

The collected samples were named “soil samples without irrigation” (SWI). Ultimately, we split each sample into thirds for 30 SWI samples. Six soil samples irrigated with WWF (SWWF) were obtained from sections P1–P3, six soil samples irrigated with RW (SRW) were collected from regions P4–P6, and six soil samples irrigated with TWE (STWE) were collected from sections P7–P9. There were 18 samples taken from irrigated soil and ten from unirrigated soil. We split each sample into thirds; thus, we tested HMs content in 84 samples.

#### 2.3.2 Plant sampling

Five samples were collected from each variety of vegetable cultivated with a specific irrigation technique. As a result, 45 vegetable samples were gathered and transported to the laboratory owing to the presence of three irrigation treatments and three varieties of vegetables. Each collected sample was examined in three duplicates, yielding 135 (3*45) vegetable samples with HMs contents assessed. Their leaves were evaluated for Coriander and Basil vegetables, while their leaves and tubers were blended for Radish vegetables before being analyzed.

#### 2.3.3 Soil sampling

The obtained soil samples were dried thoroughly in an oven before being put through a 2 mm filter to eliminate grit and other potential contaminants. In order to digest soil samples, the first 2 g of dried soil were poured into a volumetric flask with a capacity of 25 mL, and then 15 mL of nitric acid with a concentration of 4 N were added and thoroughly mixed. After that, the volumetric flask was sealed and placed in a water bath heated to 80° Celsius for 12 h. Then, the flasks were removed from the water bath and allowed to cool at the laboratory temperature. After the samples were cooled, they were filtered in a second 25 ml volumetric flask using a 42 m Whatman filter paper. The resulting extract was mixed with deionized distilled water until the desired volume was achieved [8,[18], [19], [20]].

### 2.4 Determination of heavy metals in soil

After the soil samples were prepared, the inductively coupled plasma optical emission spectrometry (ICP-OES) model was used to determine the concentration of heavy metals in each sample (SPECTRO, Germany). For the metals As, Cd, Pb, Cu, Fe, Zn, Cr, Mn, and Ni, the device's limit of detection (LOD) was 0.179, 0.049, 0.166, 0.306, 0.160, 0.270, 0.564, 0.325, 0.240 parts per billion (ppb), respectively. Also, the recovery percentage for the mentioned metals was 96.8 ± 7.2, 98.5 ± 66.6, 94.6 ± 7.2, 104.5 ± 8.4, 97.6 ± 3.3, 101.4 ± 8.4, 95.4 ± 3.6, 98.5 ± 2.8, and 97.5 ± 6.7, respectively [21].

### 2.5 Soil pollution indices

In the current study, we used indicators that compare the amount of a particular contaminant, such as HMs, and background value in irrigated soil to describe the contamination entering the cultivation's ground. I_geo_, CF, EF, C_d_, PI, and MPI were used in this study. In addition, this study used metals in non-irrigated soil as a background value [[22], [23], [24]].

#### 2.5.1 Geoaccumulation index (I_geo_)

For the determination of I_geo_, Eq. (1) was used.

(1) $$I_{geo}=\log_{2}\left[\frac{C_{i}}{{1.5B}_{n}}\right]$$

Where;

C_i_: the soil metal content.

B_n:_ the metal background content and 1.5 is the c constant used to examine minor anthropogenic influences [25].

I_geo_ ≤ 0 indicates that the soil is unpolluted; 0 < I_geo_ ≤ 1 denotes that the soil is unpolluted to moderately polluted; 1 < I_geo_ ≤ 2 implies that the soil is moderately polluted; 2 < I_geo_ ≤3 shows that the soil is moderate to heavily polluted; 3 < I_geo_ ≤ 4 means that the soil is heavily polluted; 4 < I_geo_ ≤ 5 designates that the soil is heavy to extremely polluted; I_geo_ > 5 specifies that the soil is highly polluted.

#### 2.5.2 Contamination factor (CF) and multi-element pollution indices related to CF

The CF was calculated by Eq. (2):

(2) $$CF=\frac{C_{i}}{C_{0}}$$

CF is the ratio of the soil's metal concentration (Ci) to the background value (C0).

Four contamination categories were utilized to assess the extent of metal contamination according to Table 2 [26].

**Table 2:** Level of metal contamination.

CF range	Contamination categories
CF < 1	low contamination
1 < CF < 3 is	moderate contamination
3 < CF < 6 is; and is.	considerable contamination
CF > 6	very high contamination

Due to the limitations of a single CF index, multi-element pollution indices such as C_d_ and PI were applied to the soil assessment using Eqs. (3), (4) [27,28]. In these equations, CF^i^, CF_average_, and CF_max_ represent the CF for an individual element, the mean of CFs, and maximum CF, respectively.

(3) $$C_{d}=\sum_{i=1}^{n}{CF}^{i}$$

(4) $$PI=\sqrt{\frac{{\left({CF}_{average}\right)}^{2}+{\left({CF}_{\max}\right)}^{2}}{2}}$$

The corresponding categories for C_d_ and PI are presented in Table 3 [29].

**Table 3:** Thresholds of multi-element indices for soil quality categorization.

Class	Qualification	C_d_	PI	MPI
0	Unpolluted	C_d_ < 1.5	PI < 0.7	MPI<1
1	Slightly polluted	1.5<C_d_ < 2	0.7 < PI < 1	1 < MPI<2
2	Moderately polluted	2≤C_d_ < 4	1 < PI < 2	2 < MPI<3
3	Moderately-heavily polluted	4≤C_d_ < 8	–	3 < MPI<5
4	Severely polluted	8≤C_d_ < 16	2 < PI < 3	5 < MPI<10
5	Heavily polluted	16≤C_d_ < 32	PI > 3	MPI>10
6	Extreme polluted	C_d_ > 32	–	–

**C**_**d**_: Contamination degree, **PI**: Pollution index, **MPI**: Modified pollution index.

#### 2.5.3 Enrichment factor (EF) and modified pollution index (MPI)

The EF makes it possible to differentiate between elements that originate from anthropogenic sources and those that come from natural sources and to evaluate the level of human influence [30]. This index was calculated using Eq. (5).

Eq (5) $$EF=\frac{{\left(C_{m}/C_{Fe}\right)}_{Sample}}{{\left(C_{m}/C_{Fe}\right)}_{Background}}\;)$$

C_m_ is the metal element concentration in soil, and C_Fe_ is the iron concentration.

Numerous acceptable EF reference elements are Mn, Ti, Sc Al, Ca, or Fe [31]. In our particular instance, iron (Fe) was chosen as a reference element since it is one of the most thoroughly examined elements in the soil under consideration. EF levels near 1.0 represent the earth's crust or natural source, whereas EF values greater than 1.0 represent the anthropogenic origin, and those less than 1.0 indicate metal mobilization or reduction [32]. The five grades that are used to rank the level of anthropogenic pollution [33] are EF < 2, 2≤ EF < 5, 5≤ EF < 20, 20≤ EF < 40, and EF ≥ 40. These grades imply that there is a lack of ability to prevent pollution, moderate pollution, significant pollution, very high pollution, and extremely high pollution, respectively.

Due to the restrictions of using a single EF index for each element, multi-element pollution indices such as MPI were computed using various EF-related elements. The MPI was calculated using Eq. (6) [29].

(6) $$MPI=\sqrt{\frac{{\left({EF}_{average}\right)}^{2}+{\left({EF}_{\max}\right)}^{2}}{2}}$$

EF_average_ and EF_max_ represent the average and maximum enrichment factors, respectively. The corresponding categories for MPI are presented in Table 3 [29].

### 2.6 Control and quality assurance

Reagent blanks, duplicate samples, and standard reference material (SRM 1753a, tomato leaves) obtained from the National Institute of Standards and Technology were all included in the stringent quality control system created to validate the results (Gaithersburg, MD). Eq. (7) was used to calculate the material's percentage recovery in spiked samples:

(7) $$RR\;\left(\%\right)=\frac{C_{found}-C_{real}}{C_{added}}\times 10$$

Where Cfound is the total concentration of the metal after the addition of a known amount of standard in the real sample, Creal is the original concentration of the metal in a real sample, and Cadded is the concentration of the known amount of standard that was spiked into the real sample [34].

### 2.7 Ecological risk assessment

The RI initially presented by Hakanson et al. (1980) was applied to quantify the RI of heavy metals in soil [26]. The RI combined heavy metal concentrations with environmental, ecological, and toxicological impacts. Eqs. (8), (9) are used to calculate them.

(8) $$E_{r}^{i}=T_{r}^{i}\times \frac{C_{i}}{C_{0}}$$

(9) $$RI=\sum_{i=1}^{\infty }\left(T_{r}^{i}\times \frac{C_{i}}{C_{0}}\right)=\sum_{i=1}^{\infty }E_{r}^{i}$$

Where;

C_i_: The element concentrations in a soil sample.

C_0:_ The element concentrations in the background soil.

$T_{r}^{i}$: The toxic response factor for a given metal (Cu=Pb = Ni = 5, Zn = 1, Cr = 2, Cd = 30, Mn = 1, As = 10);

$E_{r}^{i}$: The potential ecological risk factor of each metal

RI: the potential ecological risk index of all the heavy metals in the soil.

The RI of heavy metals was classified according to five levels [26], as presented in Table 4.

**Table 4:** Classification of potential ecological Factors ($E_{r}^{i}$) and risk (RI).

Assessment criterion	levels				
	Low	Moderate	Considerable	High	Very high
$E_{r}^{i}$	<40	40–80	80–160	160–320	>320
RI	<150	150–300	300–600	>600	–

**Table 5:** Descriptive parameters related to the evaluation of heavy metals in the soil of the cultivation site based on different stages of irrigation.

Irrigation	HMs	N	Amount (mg/kg)				Agricultural soil contamination standards		
							European Union	Iran	
			Mean	SD	Min	Max		pH < 7	pH > 7
Before irrigation	Fe	30	21.614	3.249	15.068	25.268	-	-	-
	Zn	30	3.236	0.951	2.192	5.039	300	200	500
	Mn	30	3.033	0.793	1.857	4.733	-	-	-
	Cu	30	1.046	0.173	0.790	1.284	140	100	200
	As	30	0.002	0.001	0.001	0.005	20	18	40
	Pb	30	0.902	0.026	0.853	0.943	300	50	75
	Cd	30	0.002	0.002	0.001	0.007	3	1	5
	Cr	30	0.003	0.001	0.002	0.006	150	110	110
	Ni	30	0.039	0.005	0.032	0.049	75	50	110
Irrigation with RW	Fe	12	95.655	12.878	73.642	108.719	-	-	-
	Zn	12	58.204	4.963	49.419	66.458	300	200	500
	Mn	12	33.074	4.382	27.771	40.254	-	-	-
	Cu	12	21.105	6.168	12.659	31.781	140	100	200
	As	12	4.821	1.165	3.190	7.031	20	18	40
	Pb	12	16.585	3.498	12.476	22.463	300	50	75
	Cd	12	2.645	0.882	1.679	4.203	3	1	5
	Cr	12	16.647	6.510	8.538	28.767	150	110	110
	Ni	12	22.265	6.748	16.368	39.077	75	50	110
Irrigation with TWE	Fe	12	66.828	5.091	58.080	72.400	-	-	-
	Zn	12	24.403	1.377	22.414	26.419	300	200	500
	Mn	12	16.630	2.015	13.667	21.130	-	-	-
	Cu	12	14.095	1.266	12.695	16.232	140	100	200
	As	12	2.298	0.306	1.766	2.711	20	18	40
	Pb	12	8.139	0.688	7.216	9.390	300	50	75
	Cd	12	1.091	0.145	0.817	1.297	3	1	5
	Cr	12	9.223	1.891	5.196	12.616	150	110	110
	Ni	12	12.681	2.698	8.441	17.159	75	50	110
Irrigation with WWF	Fe	12	80.208	2.793	74.373	83.739	-	-	-
	Zn	12	69.51	3.442	63.558	76.255	300	200	500
	Mn	12	12.159	0.823	11.216	14.101	-	-	-
	Cu	12	9.113	0.843	7.773	10.846	140	100	200
	As	12	1.317	0.132	1.135	1.579	20	18	40
	Pb	12	11.26	2.491	6.268	14.352	300	50	75
	Cd	12	5.443	0.513	4.609	6.041	3	1	5
	Cr	12	5.067	0.475	4.514	5.731	150	110	110
	Ni	12	12.394	1.589	9.999	14.170	75	50	110

**N:** Number of samples, **SD:** Standard deviation, **Min:** Minimum, **Max:** Maximum.

### 2.8 Statistical analysis

All statistical analyses used in this study were conducted by SPSS 16.0.0. The mean of each heavy metal was compared between various types of irrigation water at a significant level (α = 0.05) using a one-way analysis of variance (ANOVA).

## 3 Results and discussion

### 3.1 Comparison of irrigated and non-irrigated soils in terms of heavy metals concentration

As depicted in Table 5, the concentrations of metals measured in the soil before any irrigation and cultivation of vegetables were ordered as Fe > Zn > Mn > Cu > Pb > Ni > Cr > Cd ≈ As. Moreover, the concentration of non-toxic metals (such as Fe, Zn, Mn, and Cu) was generally higher than toxic metals (such as Pb, Cd, and Ar) in SWI. Furthermore, the amount of heavy metals in the soil of the study area is less than the level of the national standard of Iran and the European Union [1,35].

**Fig. 2:**
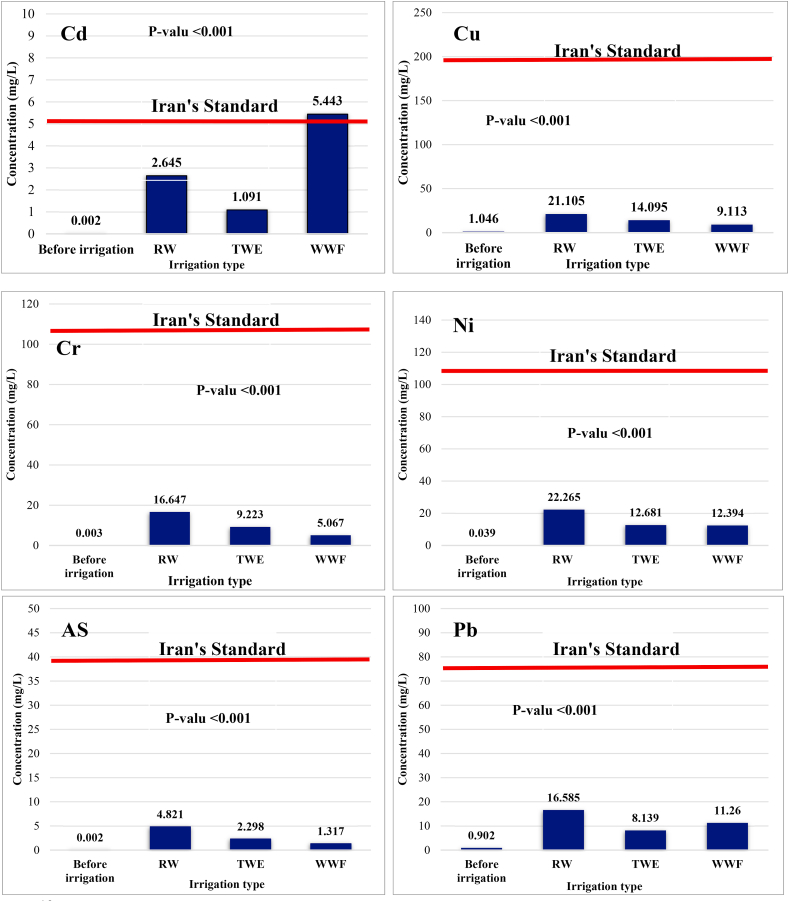

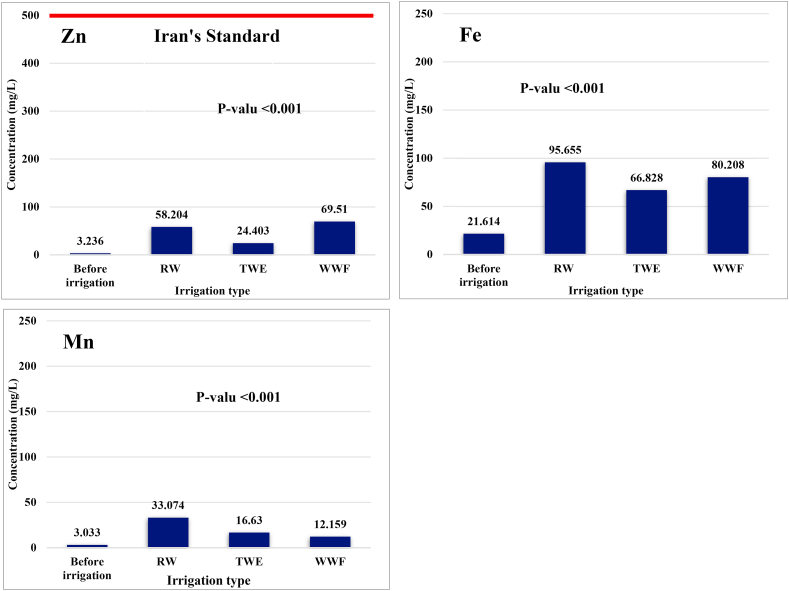
The comparison between different HMs (Cd, Cu, Cr, Ni, As, Pb, Zn, Fe, and Mn) in the soil before and after irrigation using various irrigation water sources and the Iranian government's declared national standard.

Even so, comparing the cultivation site's soil quality to national and international criteria reveals that the soil is in good condition concerning heavy metals. Fig. 2 displays a comparison between several HMs and the Iranian national standard.

**Fig. 3:**
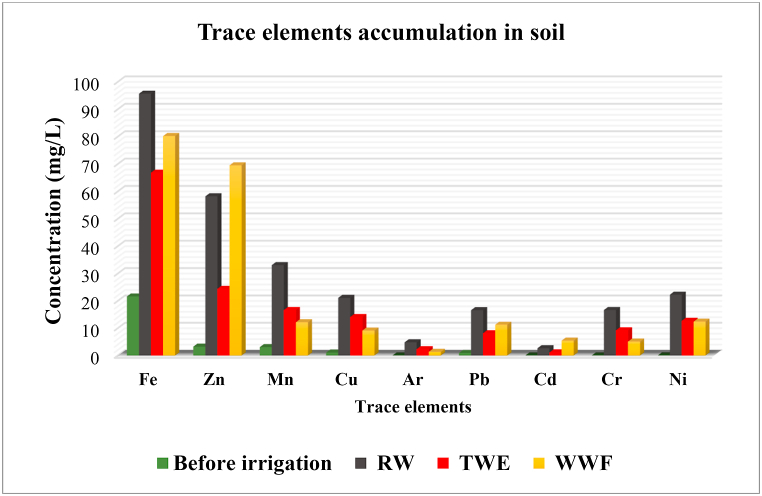
Accumulation of trace elements in irrigated soil by different irrigation methods.

Also, to summarize the effects of different irrigation methods on the accumulation of essential and unnecessary heavy metals, they are shown in Fig. 3.

Generally, the highest rate of changes was related to heavy metals cadmium, chromium, and nickel. Before irrigation, the cadmium concentration in the soil was 0.022 mg/L, and after irrigation with RW, TWE, and WWF, the concentration increased to 2.645, 1.091, and 5.443, respectively. In other words, irrigation with RW, TWE, and WWF caused the cd concentration to increase by 1322, 545, and 2721 times, respectively. Regarding RW, one of the main reasons for this increase is the many industrial units around the *Gharasoo* river that discharge effluents into the river. The changes in other heavy metals can be seen in Table 5.

The order of concentrations of the measured metals in the soil of the irrigation site irrigated with river water (SRW) is Fe > Zn > Mn > Cu > Pb > Ni > Cr > As > Cd. In the soil irrigated with treated wastewater (STWE), the order of HMs from high to low concentration was in the form of Fe > Zn > Mn > Cu > Ni > Cr > Pb > As > Cd. Whereas soil irrigated with well water and chemical fertilizer (SWWF), the order of HMs was according to Fe > Zn > Ni > Mn > Pb > Cu > Cd > Cr > As, which the obtained trend was more significantly different from the SRW and STWE [36].

The results showed that in SWI, non-toxic metals were generally higher than toxic metals. Still, after irrigation with different water sources, the concentration of HMs and their order in three types of irrigated soil were changed, as in STWE compared to SWI, the Cr and Ni concentrations were higher than Pb. In SWWF, however, the concentration of Ni was greater than Mn, the concentration of Pb was higher than Cu, and the concentration of Cd was higher than Cr. This is a probable cause of changes in heavy metal concentrations in the soil compared to before irrigation due to contamination of irrigation water sources, including river water, treated wastewater, and the high amount of heavy metals in the chemical fertilizer used in SWWF [37,38].

Our results showed that the effectiveness of irrigation resources in transferring all heavy metals to the soil is TWE < WWF < RW. The probable and foremost reason for this could be the higher pollution level of river water compared to treated wastewater. Numerous heavy metals will contaminate the Gharasoo river in Kermanshah since it is the primary recipient of all treated and occasionally untreated industrial effluent. The river may receive raw household wastewater released along the course and upstream, while raw domestic wastewater entering the treatment facility is treated, and its heavy metals are decreased [39,40].

Long-term irrigation with river water, untreated or raw wastewater, and the continuous application of chemical fertilizers to the soil can upset soil metal balance, leading to increased toxic metals such as Pb, Cd, and Ar than the standard. For example, Bahmanyar et al. (2008) stated in their study that wastewater irrigation transports more heavy metals to agricultural products' soil than ordinary water [37]. In addition, Flores-Magdaleno et al. (2011) showed that the accumulation of heavy metals in the soil had grown dramatically as a result of using wastewater for irrigation over an extended time, with the order of increasing accumulation of elements being Pb > Ni > Cd > Cr [41]. The results of studies by Al-Lahham et al. (2007) and Kalavrouziotis et al. (2012) have also shown that the use of untreated and treated wastewater for irrigation can increase soil heavy metals contents [36,38,42].

Regarding Fe and Pb content in the measured soils, from lowest to highest concentration is SRW > SWWF > STWE > SWI, while in terms of Zn and Cd, the above order was estimated as SWWF > SRW > STWE > SWI. The above order was obtained for Mn, Cr, Ni, Cu, and As as SRW > STWE > SWWF > SWI. Moreover, a significant difference between heavy metal levels in the soil before and after vegetable cultivation was observed in all of the processes studied (p < 0.001), and the ANOVA statistical test results confirmed this.

The mentioned results show that different irrigation treatments have been effective in upsetting the initial balance of soil heavy metals before planting, but the role of each treatment in changing the concentration of studied metals has been different. Based on the results, irrigation with RW followed by WWF has a more significant role than other irrigation sources in increasing Ar, Pb, and soil Cd. Since various industrial wastewater in treated and untreated (containing high levels of As and Pb) may enter the Gharasoo River (irrigation water source) [43]. Therefore, when irrigating the cultivation site with this type of irrigation source, the metals mentioned can be transferred to the soil and contaminate the soil. Similar studies have reported the same facts. Aydinalp et al. (2005) reported in their research that irrigation with water contaminated with industrial wastewater would increase the amount of Cr, Cd, Ni, and Pb in farm soils [44].

Different investigations have indicated that chemical fertilizers may contain excessive heavy metals. Therefore, overuse of fertilizers can increase metals like Cd, Pb, and Zn in the soil, and agricultural products are grown [37,[45], [46], [47], [48]]. Moreover, Rattan et al. (2005) found that high level of heavy metals such as Fe, Zn, Cu, and Pb in the irrigation water source increases metals in the soil [49]. Pourmoghadas and Zafarzadeh (2017) measured Cd, Zn, and Pb levels in Iran's three most widely used chemical fertilizers. They discovered that diammonium phosphate, triple superphosphate, and macro-granular fertilizers had Cd levels of 1.25, 1.7, and 1.5, respectively, and for Zn was 1.5, 2, and 7, respectively, which were all over the maximum concentration that is permitted for these metals in chemical fertilizers [50].

According to the findings, the concentration of heavy metals in the soil of our study area (either prior to or after farming) was lower than the level set by Iran and the standard set by the EU [35]. Although heavy metals concentration in the soil had good quality even after irrigation, long-term irrigation with river water and well water and the continuous addition of chemical fertilizers to the soil can reduce the soil quality in the cultivation place in the long run.

### 3.2 Evaluation of soil pollution

Based on the obtained results illustrated in Table 6, the I_geo_ f or all nine metals, the quality of the three types of irrigated soil falls between uncontaminated and moderately contaminated.

**Table 6:** The geoaccumulation index (I_geo_) amount and its qualification for various irrigated soils.

HMs	I_geo_					
	SRW		STWE		SWWF	
	Amount	Qualification	Amount	Qualification	Amount	Qualification
Fe	0.64	UC-MC	0.96	UC-MC	0.77	UC-MC
Zn	0.28	UC-MC	0.43	UC-MC	0.26	UC-MC
Mn	0.35	UC-MC	0.53	UC-MC	0.71	UC-MC
Cu	0.27	UC-MC	0.32	UC-MC	0.39	UC-MC
As	0.09	UC-MC	0.10	UC-MC	0.11	UC-MC
Pb	0.28	UC-MC	0.39	UC-MC	0.33	UC-MC
Cd	0.10	UC-MC	0.12	UC-MC	0.09	UC-MC
Cr	0.08	UC-MC	0.09	UC-MC	0.10	UC-MC
Ni	0.12	UC-MC	0.13	UC-MC	0.13	UC-MC

**SRW:** Soil irrigated with river water, **STWE:** Soil irrigated with treated wastewater effluent, **SWWF**: Soil irrigated with well water and fertilizer, **UC-MC:** Uncontaminated to moderately contaminated.

However, the CF was rated as “considerable contamination” for Fe metal in all three soil types and Mn metal in STWE and SWWF, while it was assessed as “very high contamination” for other metals in all three soil types (Table 7). In addition, the results showed that the two factors, C_d_ and PI, which are estimated based on CF and include all heavy metals, indicate that C_d_ for all three types of irrigated soil is classified as “extremely polluted,” while PI was classified as “heavily polluted” for all three soil types (Table 7).

**Table 7:** The contamination factor (CF) amount and its qualification for various irrigated soils.

HMs	CF					
	SRW		STWE		SWWF	
	Amount	Qualification	Amount	Qualification	Amount	Qualification
Fe	4.4	CC	3.1	CC	3.7	CC
Zn	18.0	VHC	7.5	VHC	21.5	VHC
Mn	10.9	VHC	5.5	CC	4.0	CC
Cu	20.2	VHC	13.5	VHC	8.7	VHC
As	2410.5	VHC	1149.0	VHC	658.5	VHC
Pb	18.4	VHC	9.0	VHC	12.5	VHC
Cd	1322.5	VHC	545.5	VHC	2721.5	VHC
Cr	5549.0	VHC	3074.3	VHC	1689.0	VHC
Ni	570.9	VHC	325.2	VHC	317.8	VHC
**Contamination degree (C**_**d**_**), Pollution index (PI)**						
C_d_	9924.8	EXP	5132.6	EXP	5437.2	EXP*****
PI	4000.5	HEP	2211.0	HEP	1268.4	HEP

**CC:** Considerable contamination, **VHC:** Very high contamination, **Cd:** Contamination degree, **PI:** Pollution index.

**EXP:** Extreme polluted.

The results showed that based on the estimation of EF, the contamination rate of all three soil types in terms of Fe metal was “minimal contamination,” while Cu and Pb metals in all three soil types were assessed as “moderate contamination.” The level of Mn contamination in STWE and SWWF was evaluated as “minimal contamination” while in SRW, as “moderate contamination.” EF evaluation for Zn metal showed a “moderate contamination” quality level in STWE and SRW soils, while it had a “significant contamination” level in SWWF. EF for other metals in different soils had “very high contamination” levels (Table 8). The results showed that MPI, which is estimated based on EF and considers all heavy metals, showed that MPI has a “heavily polluted” stratum for all three types of irrigated soil (Table 8).

**Table 8:** The enrichment factor (EF) amount and its qualification for various irrigated soils.

HMs	EF					
	SRW		STWE		SWWF	
	Amount	Qualification	Amount	Qualification	Amount	Qualification
Fe	1.0	MIC	1.0	MIC	1.0	MIC
Zn	4.1	MOC	2.4	MOC	5.8	SIC
Mn	2.5	MOC	1.8	MIC	1.1	MIC
Cu	4.6	MOC	4.4	MOC	2.3	MOC
As	544.7	VHC	371.6	VHC	177.4	VHC
Pb	4.2	MOC	2.9	MOC	3.4	MOC
Cd	298.8	VHC	176.4	VHC	733.4	VHC
Cr	1253.8	VHC	994.3	VHC	455.1	VHC
Ni	129.0	VHC	105.2	VHC	85.6	VHC
**Modified pollution index (MPI)**						
MPI	903.9	HEP	715.1	HEP	531.2	HEP

MIC: Minimal contamination, MOC: Moderate contamination, SIC: Significant contamination, VHC: Very high contamination, SRW: Soil irrigated with river water, STWE: Soil irrigated with treated wastewater effluent, SWWF: Soil irrigated with ell water and fertilizer, MPI: Modified pollution index, HEP: Heavily polluted.

In the present study, considering the soil pollution indices that consider only one metal (such as I_geo_, CF, and EF), in general, it can be said that the various irrigation sources studied in this study include TWE, RW, and WWF had a lower to moderate effect on the addition of iron, zinc, manganese, copper and lead to the soil. In contrast, other metals (e.g., As, Cd, Ni, and Cr) had a very high effect. Based on the finding of calculating multi-element pollution indices, it was found that the contamination of soils irrigated with heavy metals by all three irrigation sources is “high and significant contamination.” However, RW is more influential than the other two sources. Therefore, it can be said that all three irrigation sources have a high potential to contaminate the soil of vegetable crops with heavy metals.

Regarding soil contamination with metals, the prospect of irrigation sources varies since, in terms of the C_d_ index, it was RW > WWF > TWE, while in terms of PI and MPI indices, it is RW > TWE > WWF. Therefore, it can be concluded that irrigation resources used to cultivate edible vegetables in Kermanshah should be thoroughly considered and monitored. It is recommended that the water from the Gharasoo River be avoided as much as possible for irrigating vegetable crops. The entrance of raw or treated municipal and industrial wastewater into the Gharasoo River along its route is one possible reason for increased heavy metal pollution.

Our findings are compatible with certain earlier investigations but not with others. According to Barakat et al. (2020), the mean levels of various metal elements in agricultural soils, including Cd, Cr, Cu, Pb, Zn, and Fe, were higher than the local background values, except for Pb. The Igeo, CF, and EF indices revealed moderate to high heavy metal soil contamination [22]. According to the Kang et al. (2020) study in China, the I_geo_ indices of Cd and As indicated moderate pollution, whereas based on C_0_ indices, Cu and Cr stated that they were practically unpolluted. The PI demonstrated that the entire research region was prone to low pollution levels, but Yongcun and Zhaoan regions are in a pollution warning line area [51]. According to I_geo_ data collected by Qi et al. (2020) in China, Shanxi's soil was contaminated by Cd and Hg to varying degrees ranging from moderate to heavy pollution [24]. In a study by Kuerban et al. (2020) in Urumqi, China, the outcomes of I_geo_ stated that Hg moderately contaminated the analyzed farmland topsoil, while other selected elements such as Cd, As, Pb, Ni, Zn, Cu, and Cr were found to be non-contaminated [52]. Southwest Nigerian researchers, Kolawole et al. looked into heavy metal pollution and ecological risk assessment of soils from an industrial location. The findings revealed that the analyzed CF, EF, and I_geo_ confirmed high Pb, Cd, and Cu pollution in almost all tests [27].

When many research results are compared to one another and the current study, it is concluded that heavy metals such as Ar, Cd, Pb, and Hg have higher concentrations than background non-toxic metals such as Mn and Zn. Furthermore, the minimal variations in soil pollution indices between studies are due to metal concentrations in contaminated and background soil.

### 3.3 Ecological risk assessment

Our results revealed that the potential ecological risk factor ($E_{r}^{i}$) of Zn and Mn metals was less than 40 in all three types of irrigated soils, indicating that the quality of those soils was “low” in terms of these two metals. In all three types of soil, the value of $E_{r}^{i}$ was higher than 40 for other metals. $E_{r}^{i}$ was rated as “moderate” for Cu and Pb in STWE and SWWF, “considerable” in SRW, and “very high” for other metals in all three soil types based on the qualitative classification (Table 9). According to our findings, RI, which is estimated based on $E_{r}^{i}$ and considering all heavy metals, showed that RI has a “high risk” category for all three types of irrigated soil (Table 9).

**Table 9:** Different irrigated soils' possible environmental risk factors and index.

HMs	Potential ecological risk factors ($E_{r}^{i}$)					
	SRW		STWE		SWWF	
	Amount	Qualification	Amount	Qualification	Amount	Qualification
Zn	18.0	Low	7.5	Low	21.5	Low
Mn	10.9	Low	5.5	Low	4.0	Low
Cu	100.9	Considerable	67.4	Moderate	43.6	Moderate
As	24105.0	Very high	11490.0	Very high	6585.0	Very high
Pb	91.9	Considerable	45.1	Moderate	62.4	Moderate
Cd	39675.0	Very high	16365.0	Very high	81645.0	Very high
Cr	11098.0	Very high	6148.7	Very high	3378.0	Very high
Ni	2854.5	Very high	1625.8	Very high	1589.0	Very high
**Potential ecological risk index (RI)**						
RI	77954.2	High	35755.0	High	93328.4	High

SRW: **Soil irrigated with river water,** STWE**: Soil irrigated with treated wastewater effluent,** SWWF**: Soil irrigated with well water and fertilizer**.

All metals, except Cd, $E_{r}^{i}$ have values lower than 40, according to Barakat et al. (2020), indicating that these elements do not represent unacceptable ecological concerns. According to the RI results, all soil samples pose a significant ecological risk, showing values ranging from 184.49 to 292.46, averaging 239.42.

On the other hand, Cd had $E_{r}^{i}$ values ranged from 150.97 to 261.29, representing a high ecological risk. While in this study, the RI value is 77954.2, 35755.0, and 93328.4 in SRW, STWE, and SWWF, respectively; this is much higher [22]. According to Kang et al. (2020) study, the RI of paddy fields in Fujian province, China, falls within the low-risk category [51]. According to Barakat et al. (2020), Cd detected in concentrations above the local reference content plays a significant role in RI values [22]. In the current investigation, the metals As, Cd, and Cr had the highest percentage of RI values. On the other hand, cadmium should be given more attention because it contributes the most to the possible environmental issues in the province of Fujian (Er = 25.09) [51]. Qi et al. presented a projected ecological risk evaluation of Shanxi agricultural soil. They discovered that 6% of all samples had a moderate risk (150 = RI < 300), 58% had a significant risk (300 = RI < 600), 33% had an extremely high risk (600 = RI < 1200), and 3% had dangerous risk (≥1200) [24]. In research by Kuerban et al. (2020) in Urumqi, China, the mean RI in the topsoil was 259.89, suggesting a moderate ecological risk, with Hg and Cd accounting for Hg and Cd 88.87% of the RI [52]. Kolawole et al. (2018) found that the estimated PI and MPI confirmed that all soil and sediment samples from an industrial location in southern Nigeria were highly polluted [27].

A comparison of the findings of multiple investigations reveals that the variation in RI levels seen in different experiments was connected to the quantity of the examined metals in the contaminated soil. Moreover, in most similar studies in the past as well as in the present study, a considerable portion of the estimated potential ecological risk is related to heavy metals such as As, Cd, Pb, and Hg, while others such as Fe, Mn, and Zn have minimal risk.

## 4 Conclusion

Our results indicated that among the source of irrigation water for growing vegetables, the water of the *Gharasoo River* could cause higher contamination transfer to the soil than the other two sources. The main reason is that the river has become more contaminated with heavy metals since it is the major receiver of various municipal and industrial wastewaters, whether untreated or treated. Although groundwater with chemical fertilizers for vegetable growing is a healthy and reliable source from the point of view of public health and community health, chemical fertilizers can increase heavy metals in the soil of the cultivation region and have high economic costs. Therefore, it can be concluded that the best type is treated domestic wastewater among the three irrigation water sources studied. Thus, if the quality of treated wastewater is under national and international standards, in addition to providing the water needed for irrigation, it can also reduce the needs of farmers in terms of chemical fertilizers because the nutrients in wastewater can be an excellent alternative to fertilizers. Therefore, in this regard, treated wastewater has excellent economic efficiency compared to the other two sources. In the present study, considering the soil pollution indices that consider only one metal, in general, it can be said that the various irrigation sources studied in this study, including well water with chemical fertilizers, treated wastewater effluent, and river water, the addition of iron, zinc, manganese, copper, and lead to the soil had a little to moderate effect.

In contrast, this effect was very high for other metals (e.g., As, Cd, Ni, and Cr). Based on the results of calculating multi-element pollution indices, it was found that the contamination of soils irrigated with heavy metals by all three irrigation sources is “high and significant contamination.” However, river water is more influential than the other two sources. Therefore, it can be said that all three irrigation sources have a high potential to contaminate the soil of vegetable crops with heavy metals. Furthermore, the results revealed that the risk index for all three types of irrigated soil has a high-risk category, so it can be concluded that irrigation resources used to grow edible vegetables in Kermanshah should be monitored closely. As much as possible, the Gharasoo River's water should not be used for vegetable irrigation.

## Declarations

### Author contribution statement

Hamed Soleimani: Conceived and designed the experiments; Performed the experiments; Wrote the paper. Borhan Mansouri and Amir Kiani: Conceived and designed the experiments; Analyzed and interpreted the data. Abdullah Khalid Omer; Gholamreza Ebrahimzadeh: Contributed reagents, materials, analysis tools, or data; Wrote the paper. Moslem Tazik: Analyzed and interpreted the data; Contributed reagents, materials, analysis tools or data. Kiomars Sharafi: Conceived, and designed the experiments; Contributed reagents, materials, analysis tools, or data.

### Funding statement

This work was supported by the Research Council of 10.13039/501100005317Kermanshah University of Medical Sciences for financial assistance (Grant Number: 990606).

### Data availability statement

Data included in article.

## Declaration of interest's statement

The authors declare no conflict of interest.

### Additional information

No additional information is available for this paper.
